# Die Zahnheilkunde, nach ihrem neusten Standpunkte, ein Lehibuck für Zahraerzte unt Aerzte

**Published:** 1853-01

**Authors:** 


### BIBLIOGRAPHICAL.

*Die Zahnheilkunde, nach ihrem neusten Standpunkte, ein Lehibuck für Zahraerzte und Aerzte, von J. Liuderer, Zahnarzt in Berlin, mit* 6 *tafeln in Stahlstich.* Erlaugen, 1851.

We have already had occasion to notice the two volumes of the Handbuch by the same author. The book now before us is a general treatise on dentistry, containing a thorough and careful review of the art down to the latest moment. The anatomy, special and microscopic, of the teeth occupies a considerable portion of the book. The descriptions are minute and faithful, characterized by true German industry and precision.

Not the least attractive portion of the work is that devoted to the history and literature of dentistry. This is full of interesting and instructive particulars. The notions of various nations in regard to the teeth have been gathered from books of travels; classical literature has been ransacked for passages relating to these organs; Greek, Roman and Arabic medical works have been scrutinized in reference to ancient and medical dentistry. Paulus Ægineta, Rhazes, Avicenna and Abulkasis whose acquaintance we made when our preceptor, in our early studies, set us to read the history of medicine, are made to bear testimony to the management of these organs in their day. The progress of improvement, after those dark ages had passed away, is then traced with equal minuteness, industry and learning, and so the history is brought down to the day of publication.

In short, the book is a true German one, a monument of patient application, and exhaustive study. We recommend it, especially the latter chapters on the history of dentistry, to the perusal of our readers.

